# Supplementary material for: Structure and function of an Arabidopsis thaliana sulfate transporter
Source: Nat Commun. 2021 Jul 22;12:4455. doi: 10.1038/s41467-021-24778-2 (PMC8298490; doi:10.1038/s41467-021-24778-2)
Supplement: Supplementary file 1 — Supplementary Information [file 41467_2021_24778_MOESM1_ESM.pdf]

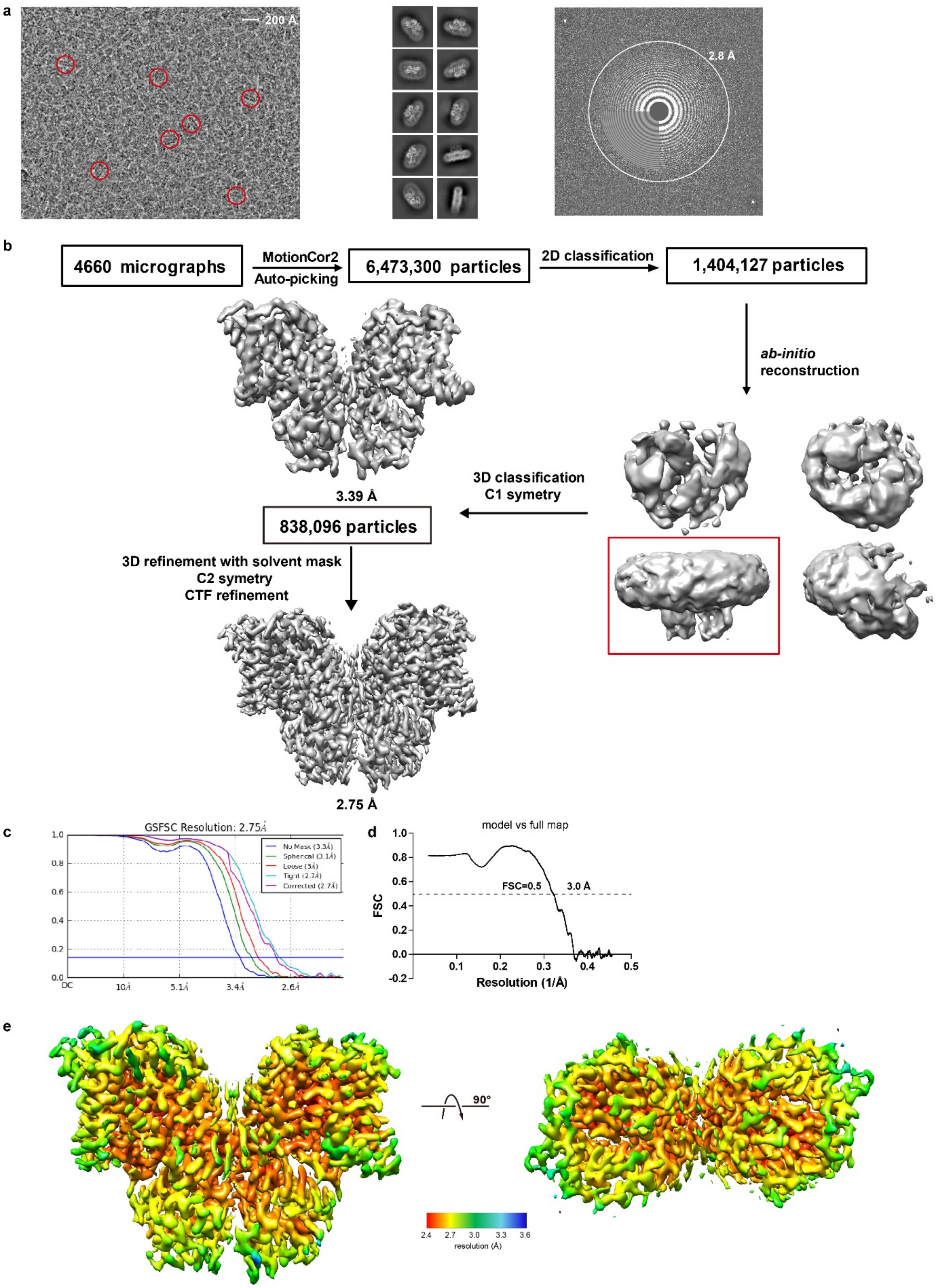
a
200 Å
2.8 Å
b
4660 micrographs
MotionCor2
Auto-picking
6,473,300 particles
2D classification
1,404,127 particles
ab-initio
reconstruction
3D classification
C1 symetry
3.39 Å
838,096 particles
3D refinement with solvent mask
C2 symetry
CTF refinement
2.75 Å
c
GSFSC Resolution: 2.75Å
No Mask (3.3Å)
Spherical (3.1Å)
Loose (3Å)
Tight (2.7Å)
Corrected (2.7Å)
1.0
0.8
0.6
0.4
0.2
0.0
DC
10Å
5.1Å
3.4Å
2.6Å
d
model vs full map
FSC
FSC=0.5
3.0 Å
Resolution (1/Å)
1.0
0.8
0.6
0.4
0.2
0.0
-0.2
0.1
0.2
0.3
0.4
0.5
e
90°
2.4
2.7
3.0
3.3
3.6
resolution (Å)

Supplementary Figure 1. Cryo-EM data processing of AtSULTR4;1. a. A representative micrograph of AtSULTR4;1 (left), its Fourier transform (right) and representative 2D class averages (middle). Representative particles are highlighted with red circles. **b.** Flow chart for data processing and the final maps of AtSULTR4;1 (**Methods**). **c.** Gold-standard Fourier shell correlation (FSC) curve for the final map shown in **b**. **d.** Fourier shell correlation curves of the atomic model of AtSULTR4;1 versus the full map. **e.** Local-resolution map of AtSULTR4;1 shown in two orientations.

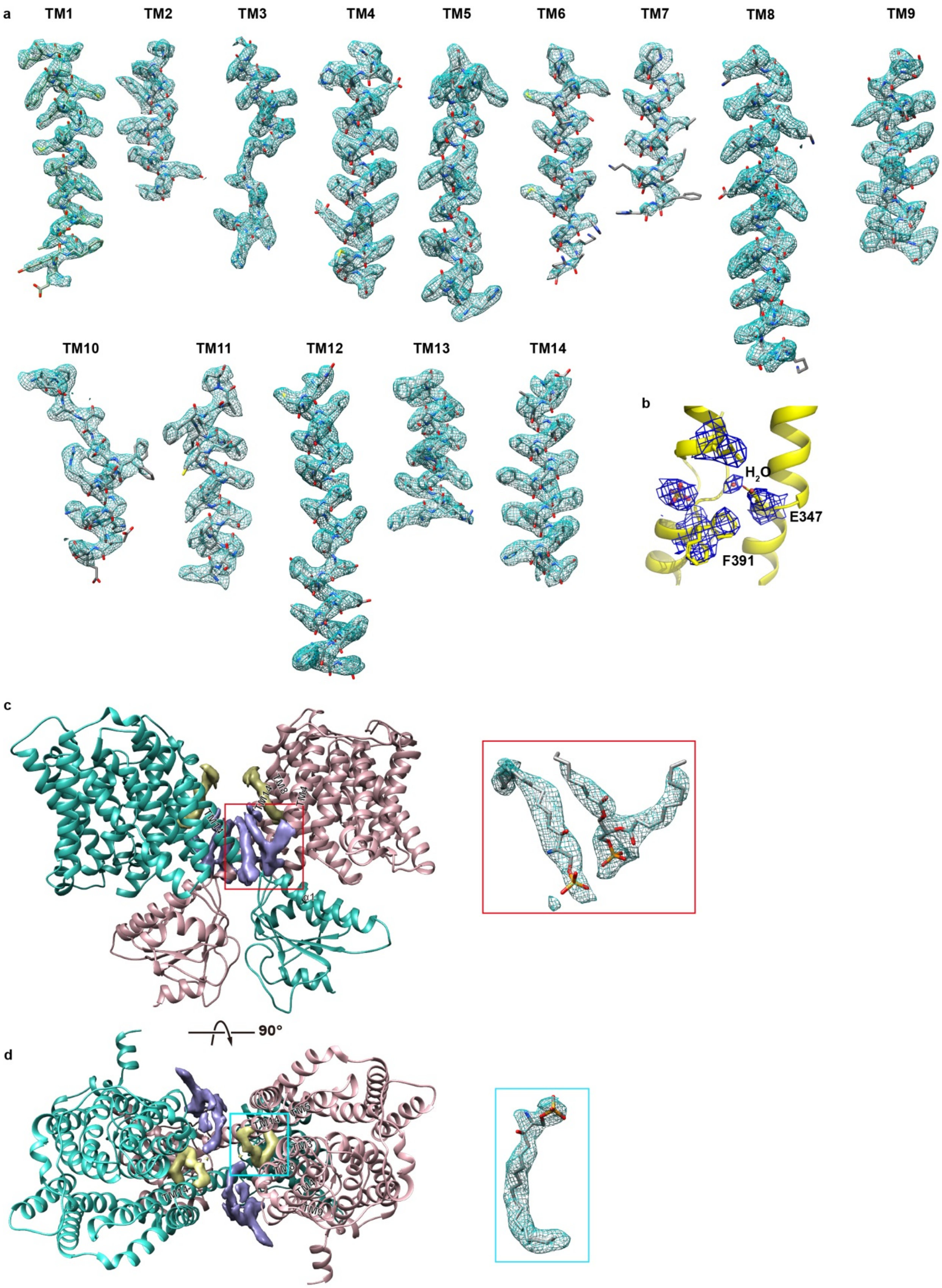

a
TM1
TM2
TM3
TM4
TM5
TM6
TM7
TM8
TM9
TM10
TM11
TM12
TM13
TM14
b
H_2O
E347
F391
c
90°
d

Supplementary Figure 2. Representative densities. a. Individual secondary structures of AtSULTR4;1 shown as sticks, contoured in their density (cyan mesh). **b.** Density map around SO_4^{2-}. **c. and d.** Lipids in the map. Lipids densities are shown in purple or yellow surfaces on the protein (left), and as blue mesh in the zoomed view (right). Putative lipids shown as sticks are fitted in the densities.

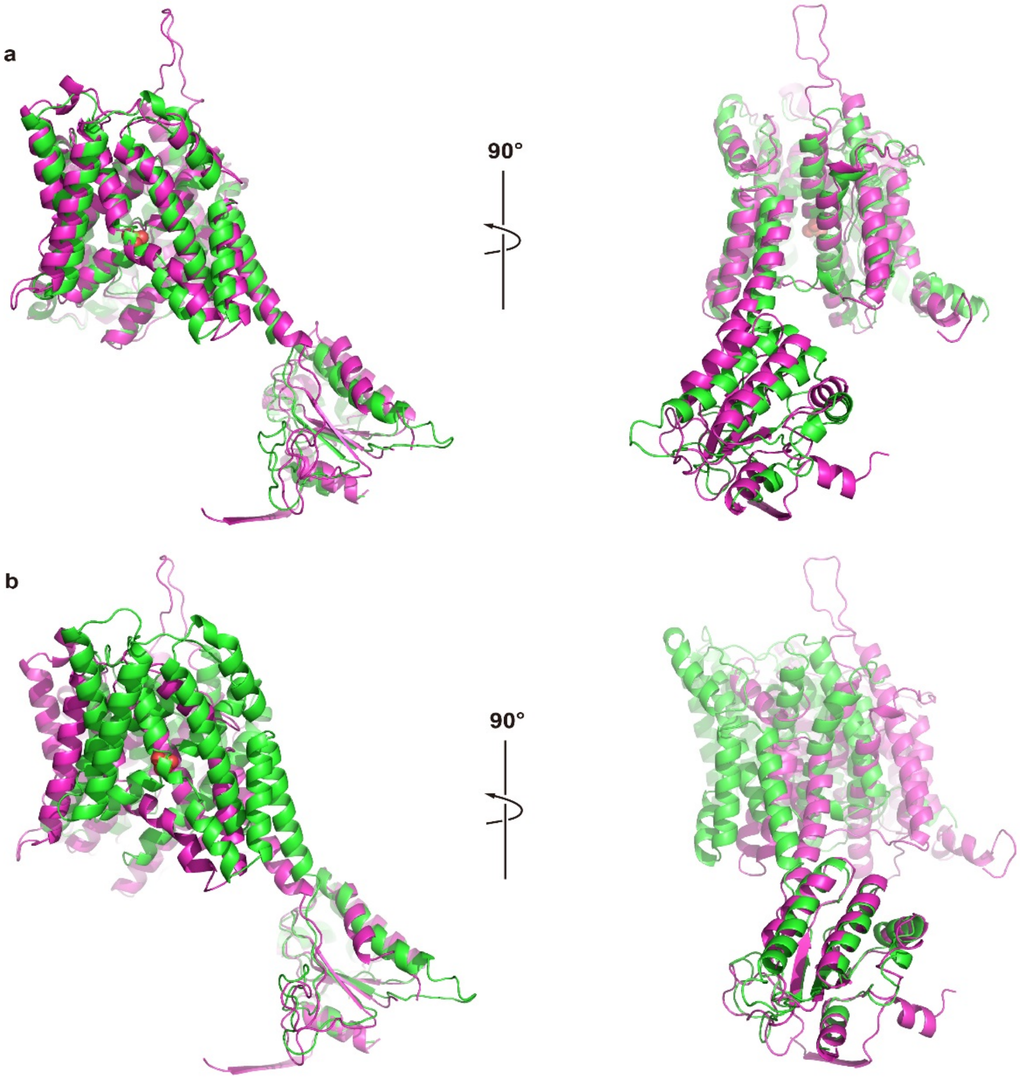

Supplementary Figure 3. Structural alignment of AtSULTR4;1 and mSLC26A9. AtSULTR4;1 (green) and mouse SLC26A9 (magenta) monomers aligned at their TM domains (**a**) or STAS domains (**b**), shown in two orientations.

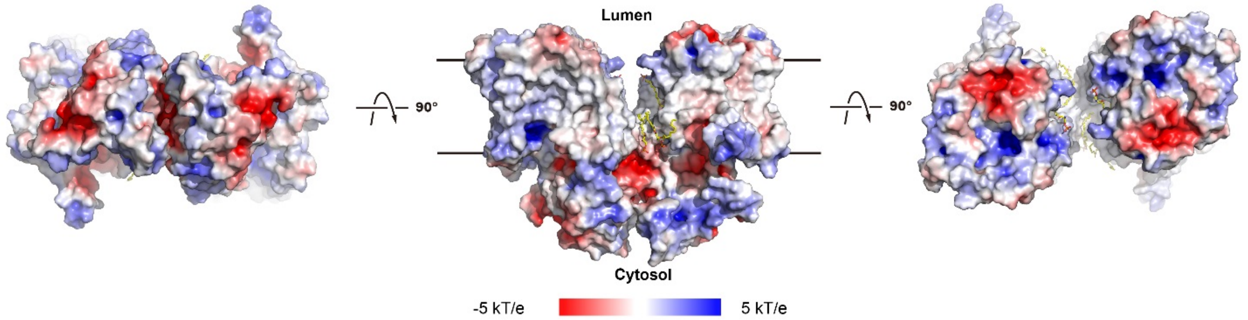

Supplementary Figure 4. Electrostatic potential map of AtSULTR4;1. Negative and positive charges are shown in red and blue, respectively. Lipids are shown as sticks.

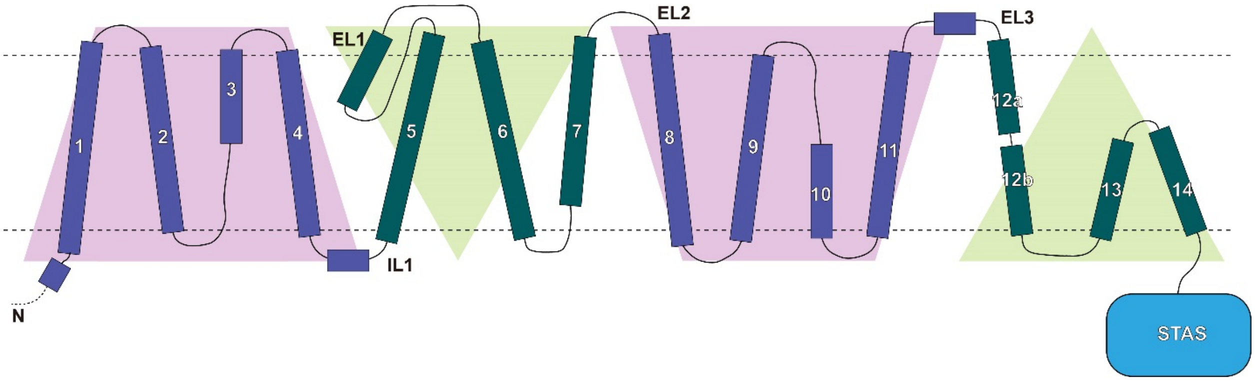

Supplementary Figure 5. Pseudo 2-fold symmetry of AtSULTR4;1 TM helices. Purple trapezoids indicate the TM helices that form the core domain, and green triangles indicate those forming the gate domain.

Group	Protein	110–119	150–156	344–350	386–397
SO_4^{2-} transport	AtSULTR4;1	VPQAMSYAKL	GPVALVS	AILESVG	PATGSFSRSAVN
	AtSULTR1;2	IPQDIGYAKL	GPVAVVS	ALTEAVA	VATGSFSRSAVN
	AtSULTR2;1	IPQSIGYATL	GPVAVVS	ALTEAIA	AATGSFSRTAVN
	AtSULTR3;1	IPQGISYAKL	GTVAVAS	ALAEGVA	LTTGPFSRSAVN
	hSLC26A11	IPQALAYAEV	GPTAIMS	GLLESIA	PVTGSFGRTAVN
	hSLC26A1	VPQAIAYSLL	GIFSLLC	AAAFSIS	ATSAALAKSLVK
	hSLC26A2	VPQSIAYSLL	GIFGVLC	GFAITVS	TTSAALAKTLVK
	hSLC26A7	VTQGLAFAVL	GTFALTS	GYVASLA	PSAAAMGRTAGL
No SO_4^{2-} transport	hSLC26A3	VLQGLAFALL	GPFPILS	AFAVAFS	AGSTALSRSAVQ
	hSLC26A4	TLQGMAYALL	GPFPVVS	AYAIAVS	VATTALSRTAVQ
	hSLC26A5	LPQGLAFAML	GPFAVIS	GFSVTIS	SISCSLSRSLVQ
	hSLC26A6	LPQGLAYALL	GTFAVMS	GFAIAIS	PVSCSMSRSLVQ
	hSLC26A8	VPQGLTLSLL	GSFFLVS	SSFLLIF	VFTGAIARTIIQ
	hSLC26A9	VPQGMAFALL	GTFAVIS	SYVINLA	VICCALSVTLAV
	SLC26Dg	IPEAIAFSII	AATGAMA	GLLESLL	AGCAMIGQSMIN
	BicA	LPMALAFGIA	EPTGPMT	GCIDALL	GGAGATMGTVVN

Supplementary Figure 6. Amino acid sequence alignment of SULTR/SLC26 homologs. Amino acid sequences of AtSULTR4;1 (RefSeq accession NM_121358.3 [https://www.ncbi.nlm.nih.gov/nuccore/NM_121358.3]), AtSULTR1;2 (NM_106449.3 [https://www.ncbi.nlm.nih.gov/nuccore/NM_106449.3]), AtSULTR2;1 (NM_121056.3 [https://www.ncbi.nlm.nih.gov/nuccore/NM_121056.3]), AtSULTR3;1 (NM_115049.5 [https://www.ncbi.nlm.nih.gov/nuccore/NM_115049.5]), human SLC26A1 (NM_022042.4 [https://www.ncbi.nlm.nih.gov/nuccore/NM_022042.4]), SLC26A2 (NM_000112.4 [https://www.ncbi.nlm.nih.gov/nuccore/NM_000112.4]), SLC26A7 (NM_052832.4 [https://www.ncbi.nlm.nih.gov/nuccore/NM_052832.4]), SLC26A11 (NM_173626.4 [https://www.ncbi.nlm.nih.gov/nuccore/NM_173626.4]), SLC26A3 (NM_000111.3 [https://www.ncbi.nlm.nih.gov/nuccore/NM_000111.3]), SLC26A4 (NM_000441.2 [https://www.ncbi.nlm.nih.gov/nuccore/NM_000441.2]), SLC26A5 (NM_198999.3 [https://www.ncbi.nlm.nih.gov/nuccore/NM_198999.3]), SLC26A6 (NM_022911.3 [https://www.ncbi.nlm.nih.gov/nuccore/NM_022911.3]), SLC26A8 (NM_052961.4

[https://www.ncbi.nlm.nih.gov/nuccore/NM_052961.4]), SLC26A9 (NM_052934.4 [https://www.ncbi.nlm.nih.gov/nuccore/NM_052934.4]), SLC26Dg (WP_011525799.1 [https://www.ncbi.nlm.nih.gov/nuccore/WP_011525799.1]), and BicA (WP_010873740.1 [https://www.ncbi.nlm.nih.gov/nuccore/WP_010873740.1]) are aligned using the Clustal Omega server[60]. Only segments responsible for SO_4^{2-} coordination on AtSULTR4;1 are shown for clarity. Residues coordinating SO_4^{2-} on AtSULTR4;1 are marked with red background. Conserved residues in other SULTR/SLC26 homologs are marked with red letters; bolded red letters indicate completely conserved residues, and regular red letters indicate similar residues.

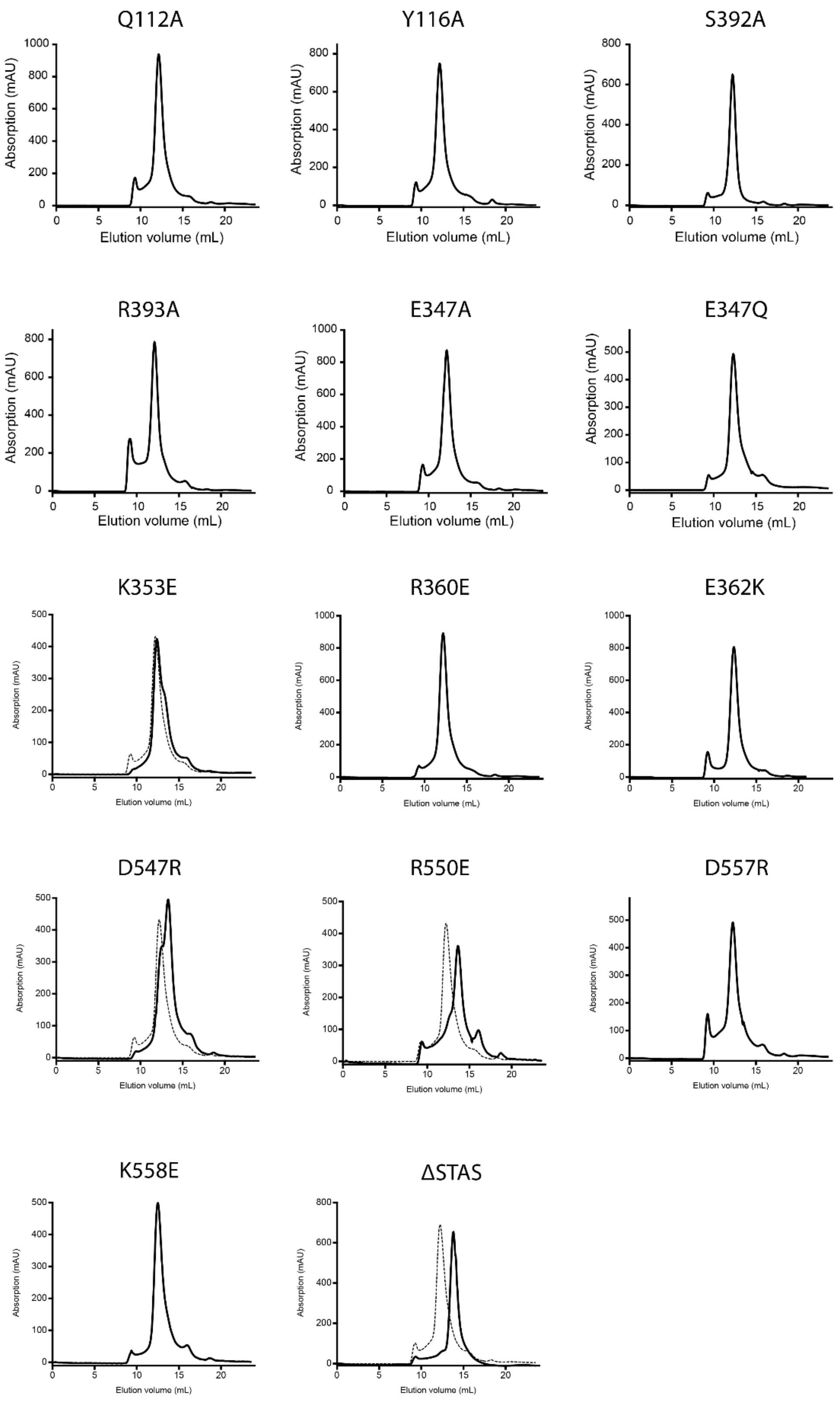
Q112A
Y116A
S392A
R393A
E347A
E347Q
K353E
R360E
E362K
D547R
R550E
D557R
K558E
ΔSTAS
Absorption (mAU)
Elution volume (mL)

Supplementary Figure 7. AtSULTR4;1 mutants. Size exclusion chromatography profiles of AtSULTR4;1 mutants. For the mutants K353E, D547R, R550E, and ΔSTAS, the profile of AtSUTLR4;1 WT is plotted as a dashed curve.

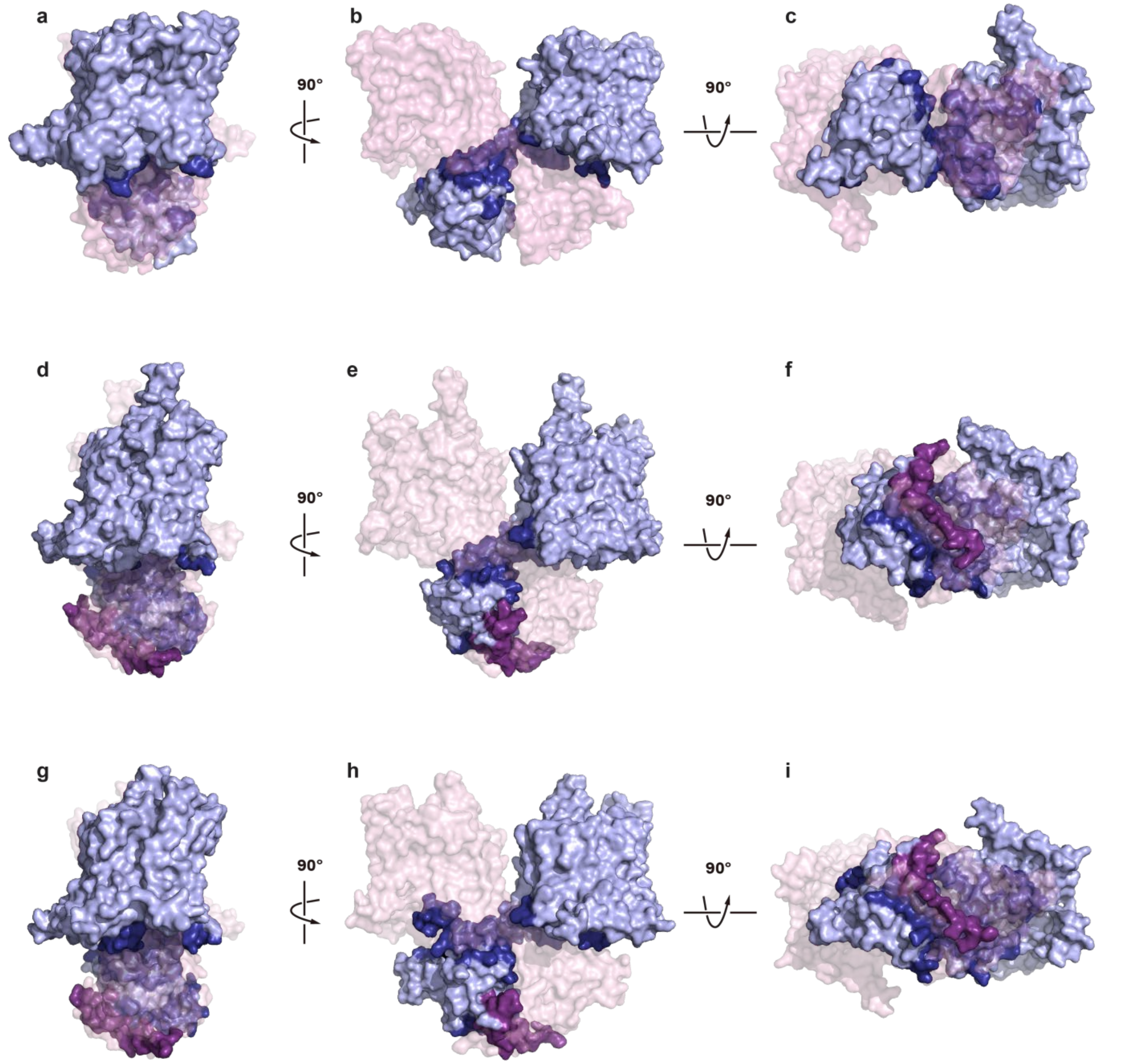

Supplementary Figure 8. Comparison of dimer interfaces in AtSULTR4;1 and SLC26A9. Structures of AtSULTR4;1 (**a-c**), mouse SLC26A9 (**d-f**), and human SLC26A9 (**g-i**) are in surface view. One monomer is translucent for better representation of the dimer interface. The dimer interface is colored deep blue. Additional dimer interfaces formed by the N-termini and the STAS domains in mouse and human SLC26A9 are colored purple.

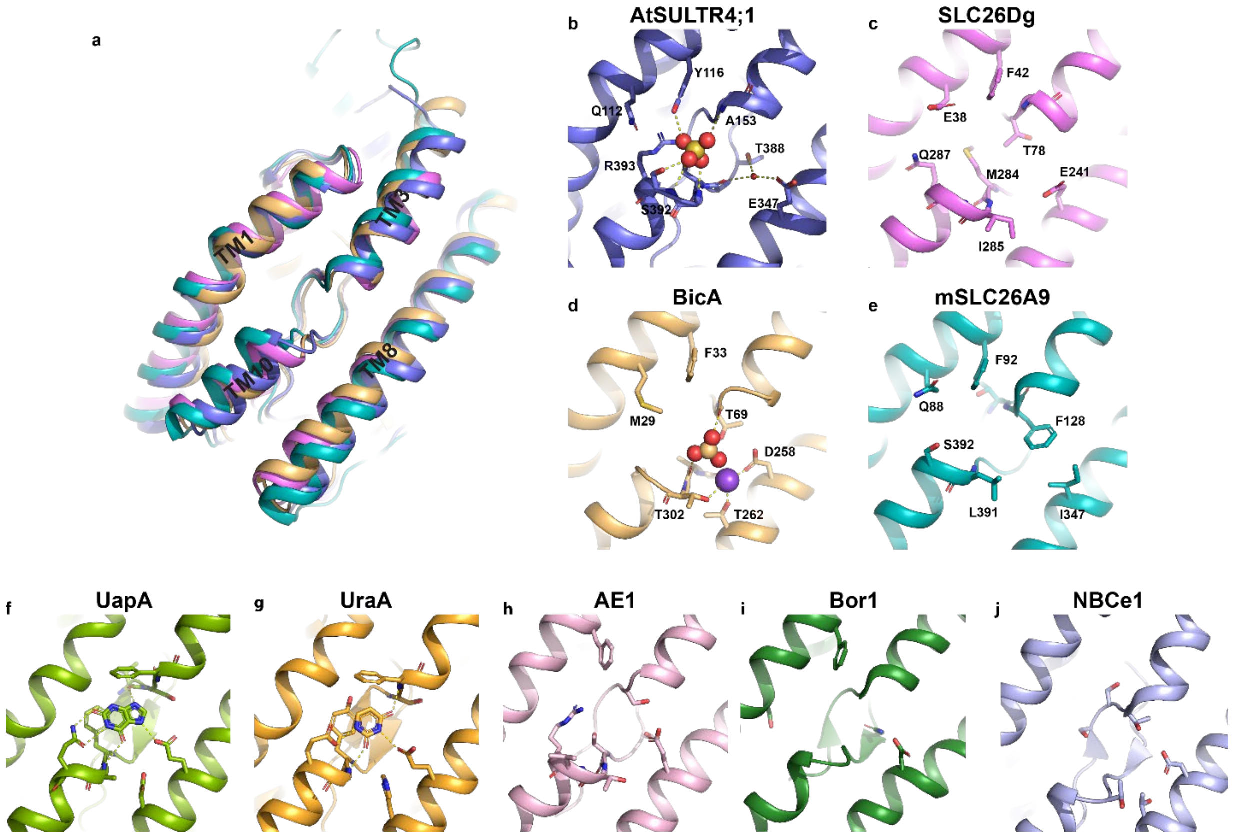

Supplementary Figure 9. Comparison of substrate binding sites in SLC26/SLC4/SLC23 homologs. a. Superimposition of the core domains from four SLC26 homologs. AtSULTR4;1, SLC26Dg (PDB ID 5DA0 [https://www.rcsb.org/structure/5DA0]), BicA (6KI1 [https://www.rcsb.org/structure/6KI1]), and mouse SLC26A9 (6RTC [https://www.rcsb.org/structure/6RTC]) are shown in violet, pink, light yellow, and teal, respectively. **b-e.** Substrate binding sites of the SULTR/SLC26 homologs. Residues that may contribute to substrate binding are shown as sticks. **f-j.** Substrate binding sites of SLC4/SLC23 homologs. The PDB IDs for UapA, UraA, AE1, Bor1, and NBCe1 are 5I6C [https://www.rcsb.org/structure/5I6C], 3QE7 [https://www.rcsb.org/structure/3QE7], 4YZF [https://www.rcsb.org/structure/4YZF], 5L25 [https://www.rcsb.org/structure/5L25], and 6CAA [https://www.rcsb.org/structure/6CAA], respectively.

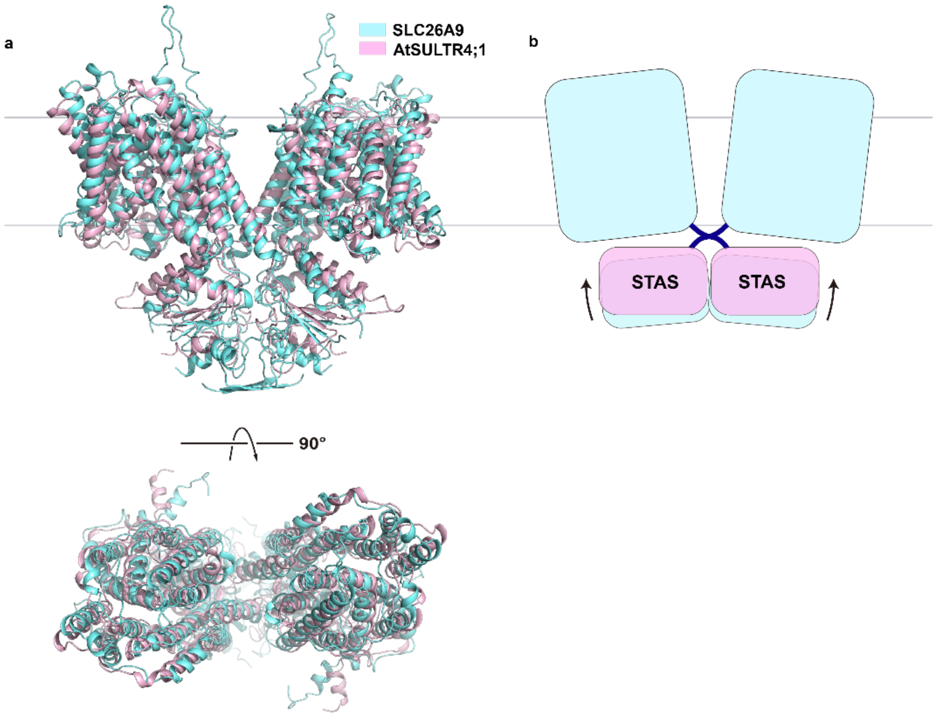

Supplementary Figure 10. Potential movement of STAS domains in transport. a. Structural alignment between AtSULTR4;1 (pink) and SLC26A9 (cyan, PDB ID 6RTC [https://www.rcsb.org/structure/6RTC]) dimers, shown in two orientations. **b.** Cartoon model of conformational changes in the STAS domain from the structural alignment in **a**.

Supplementary table 1. Summary of cryo-EM data collection, processing, and structural refinement

	AtSULTR4;1 (EMD-23351) (PDB 7LHV)
**Data collection and processing**	
Magnification	81,000
Voltage (kV)	300
Electron exposure (e–/Å^2)	50
Defocus range (μm)	[-2.0, -0.8]
Pixel size (Å)	1.08
Symmetry imposed	C2
Initial particle images (no.)	6,473,300
Final particle images (no.)	838,096
Map resolution (Å)	2.8
FSC threshold	0.143
Map resolution range (Å)	2.4-3.6
**Refinement**	
Initial model used (PDB code)	6RTC
Model resolution (Å)	3.0
FSC threshold	0.5
Model resolution range (Å)	2.4-3.6
Map sharpening *B* factor (Å^2)	-100
Model composition	
Non-hydrogen atoms	9112
Protein residues	1150
Ligands	8
B factors (Å^2)	
Protein	64.3
Ligand	66.8
R.m.s. deviations	
Bond lengths (Å)	0.004
Bond angles (°)	0.516
Validation	
MolProbity score	1.47
Clashscore	4.45
Poor rotamers (%)	0
Ramachandran plot	
Favored (%)	96.3
Allowed (%)	3.7
Disallowed (%)	0
